# Nanoemulsion containing essential oil from *Xylopia ochrantha* Mart. produces molluscicidal effects against different species of *Biomphalaria (Schistosoma* hosts*)*

**DOI:** 10.1590/0074-02760180489

**Published:** 2019-04-08

**Authors:** Fernanda de Paula Araújo, Ricardo Diego Duarte Galhardo de Albuquerque, Leonardo da Silva Rangel, Gabriel Rocha Caldas, Luís Armando Cândido Tietbohl, Marcelo Guerra Santos, Eduardo Ricci-Júnior, Silvana Thiengo, Monica Ammon Fernandez, José Augusto Albuquerque dos Santos, Robson Xavier Faria, Leandro Rocha

**Affiliations:** 1 Universidade Federal Fluminense, Laboratório de Tecnologia de Produtos Naturais, Niterói, RJ, Brasil; 2 Fundação Oswaldo Cruz-Fiocruz, Instituto Oswaldo Cruz, Laboratório de Avaliação e Promoção da Saúde Ambiental, Rio de Janeiro, RJ, Brasil; 3 Fundação Oswaldo Cruz-Fiocruz, Instituto Oswaldo Cruz, Laboratório de Toxoplasmose e Outras Protozooses, Rio de Janeiro, RJ, Brasil; 4 Universidade do Estado do Rio de Janeiro, Faculdade de Formação de Professores, Departamento de Ciências, Rio de Janeiro, RJ, Brasill; 5 Universidade Federal Fluminense, Programa de Pós-Graduação em Ciências e Tecnologia, Niterói, RJ, Brasil; 6 Universidade Federal do Rio de Janeiro, Centro de Ciências da Saúde, Departamento de Medicamentos, Rio de Janeiro, RJ, Brasil; 7 Fundação Oswaldo Cruz-Fiocruz, Instituto Oswaldo Cruz, Laboratório de Malacologia, Rio de Janeiro, RJ, Brasil

**Keywords:** essential oil nanoemulsion, Xylopia ochrantha, mollusc control, Biomphalaria, schistosomiasis

## Abstract

**BACKGROUND:**

This work describes a chemical study of the essential oil from leaves of *Xylopia ochrantha*, an endemic Annonaceae species from Brazil, and its activity against *Biomphalaria* species. Considering its poor solubility in aqueous medium, the essential oil was nanoemulsified to evaluate its action on controlling some mollusc species of genus *Biomphalaria*, snail hosts of *Schistosoma mansoni* that causes schistosomiasis, which mainly affects tropical and subtropical countries.

**OBJECTIVES:**

The main aims of this work were to analyse the chemical composition of essential oil from *X. ochrantha*, and to evaluate the effect of its nanoemulsion on molluscs of genus *Biomphalaria* and their oviposition.

**METHODS:**

Chemical analysis was performed by gas chromatography coupled to mass spectrometry. Nanoemulsions were prepared by a low energy method and characterised by particle size and polydispersity index. Biological assays evaluating the mortality of adult species of *B. glabrata*, *B. straminea* and *B. tenagophila* and their ovipositions upon contact with the most stable nanoemulsion during 24 and 48 h were performed.

**FINDINGS:**

Chemical analysis by mass spectrometry revealed the majority presence of bicyclogermacrene and germacrene D in the essential oil. The formulation with a hydrophilic-lipophilic balance (HLB) of 9.26 was the most suitable for the oil delivery system. This nanoemulsion caused the mortality in *B. tenagophila*, *B. straminea* and *B. glabarata* of different sizes at levels ranging from 50 to 100% in 48 h. Additionally, the formulation could inhibit the development of deposited eggs.

**CONCLUSION:**

Thus, these results suggest the use of nanoemulsified essential oil from *X. ochrantha* as a possible alternative in controlling some *Biomphalaria* species involved in the schistosomiasis cycle.

Schistosomiasis is an acute and chronic parasitic disease caused by trematode worms of the genus *Schistosoma* and is transmitted by several types of snails. It affects people along 78 countries mainly in tropical and subtropical regions. Schistosomiasis is the second largest infectious-parasitic disease in the world after malaria. In 2014, at least 61.6 million people in the world have been treated for schistosomiasis.^1^ The acute form of this disease causes symptoms like fever, fatigue, myalgia, malaise, non-productive cough, whereas later stages show abdominal pathologies such as diarrhoea, diffuse abdominal pain, and hepatosplenomegaly. A chronic condition occurs when *Schistosoma* deposits its eggs and reactions from the host immune system lead to urinary, intestinal, hepatic, and ectopic forms of the disease.^2^

Human schistosomiasis is caused by *Schistosoma mansoni* that uses molluscs of genus *Biomphalaria* as its intermediate host. In many cases, prevention methods like *Biomphalaria* and *Oncomelania* eradication using chemical pesticides are relevant to control the disease. Niclosamide (Bayluscide, Bayer, Leverkusen, Germany) is the only commercially available molluscicide recommended by the World Health Organization (WHO) for large-scale use in Schistosomiasis Control Programs.^3^ However, this drug is toxic to other organisms, and resistance to niclosamide requires a search for new drugs and substances to be used in the vector control.^4^ Therefore, the WHO encourages a search for alternative drugs in schistosomiasis control. Natural products can be seen as a promising alternative as they are plentiful in schistosomiasis-endemic countries and have a large number of different substances in their extracts, which hinders the appearance of vectors-resistance.^5^

Essential oils are used to the control of snails, and plant species with high yields of essential oil as from genus *Xylopia*, showed activity against *Biomphalaria glabrata*, a relevant intermediate host of *S. mansoni*.^6^ However, essential oils have low solubility in water, which may compromise their activity against *Biomphalaria.* Therefore, they must be processed into a formulation capable of viable use in biological activity studies. Nanotechnology has been used to solve problems of hydrosolubility and stability for active substances. There are several nanocarriers of drugs including nanoparticles, liposomes, and nanoemulsions (NEs).^7^

Nanoemulsions have been widely used as nanocarriers of hydrophobic drugs and essential oils. The majority of NEs are dispersions of nanometric oil droplets in water stabilized by surfactants. The droplet size of a nanoemulsion is typically in the range of 20-200 nm.^8^ ^,^ ^9^ Some key advantages of these nanocarriers are easy preparation, simple composition, the possibility of industrial-scale production, low cost of production, and high thermodynamic stability.^10^ ^,^ ^11^

*Xylopia ochrantha* Mart. is an endemic Annonaceae species from Brazil and is popularly known as “imbiú-prego”. There is little information regarding the chemical, pharmaceutical, and biological activities from this species.^12^ ^,^ ^13^ Thus, this work aimed to identify the chemical composition of essential oil from *X. ochrantha* leaves and to evaluate the activity of a nanoemulsion produced with this oil on the developmental stages (oviposition) and mortality of different *Biomphalaria* species.

## MATERIALS AND METHODS

*Plant material* - Three specimens of *X. ochrantha* Mart., Annonaceae, were collected at Restinga de Jurubatiba National Park (RJ), Brazil, in Clusia scrub vegetation (22º13’0.95’’S - 41º35’164”W, 22º13’685”S - 41º35’256”W, 22º12’420”S - 41º36’354”W) during the day on July 27, 2016. This species was identified by botanist Dr Santos MG and a voucher specimen was deposited at the Faculdade de Formação de Professores herbarium (Universidade do Estado do Rio de Janeiro, Brazil) under registration number RFFP 14.500.

*Extraction of essential oil* - A mixture of leaves (1000 g) from the three specimens collected was turbolised with distilled water. The material was then placed in a five L bottom flask and subjected to hydrodistillation for 4 h in a Clevenger-type apparatus. In the end, the oils were collected and stored at 4ºC for further analyses.

*Gas chromatography/mass spectrometry analysis* - The essential oil recovered was analysed using a GCMS-QP5000 (Shimadzu) gas chromatograph, equipped with a mass spectrometer using electron ionisation. Gas chromatographic (GC) conditions were as follows: injector temperature, 260ºC; flame ionisation detection (FID) temperature, 290ºC; carrier gas, Helium; flow rate, 1 mL/min; and split injection with split ratio 1:40. The oven temperature was initially 60ºC and was increased to 290ºC at a rate of 3ºC/min. One microliter of each sample, dissolved in dichloromethane (1:100 mg/μL), was injected into a DB-5 column (0.25 mm I.D, 30 m in length, and 0.25 μm film thickness). Mass spectrometry (MS) electron ionisation was at 70 eV and scan rate was 1 scan/s. Retention indices (RI) were calculated by extrapolating the retention times of a mixture of aliphatic hydrocarbons (C_9_-C_30_) analysed under the same conditions.^14^ Substances were identified by comparing their retention indices and mass spectra with those reported in literature.^15^ The MS fragmentation pattern of compounds was also compared with NIST mass spectra libraries. Quantitative analysis of the chemical constituents was performed by flame ionisation gas chromatography (CG/FID), under the same conditions as GC/MS analysis. Percentages of these compounds were obtained by FID peak area normalisation method.

*Nanoemulsification method and determination of hydrophilic-lipophilic balance (HLB)* - Emulsification was performed by modification of the low energy method described by Ostertag et al.^16^ Emulsions comprised 5% (w/w) of *X. ochrantha* oil, 5% (w/w) of surfactants, and 90% (w/w) water. Tween 20 and Span 80 were used as surfactants to prepare the nanoemulsions. Various emulsions with HLB values ranging from 4.3 to 16.7 were prepared by mixing the surfactants in different proportions. For preparation of nanoemulsions, oily phase constituted by *X. ochrantha* oil and surfactants was homogenised by magnetic stirring (400 rpm) for 30 min at room temperature. After this, the aqueous phase (distilled water) was added to the oily phase under the same continuous magnetic stirring (400 rpm) for 1 h. The formulations were analysed to verify their stability by analysis of droplet size and polydispersity index values. The formulation with the lowest values of droplet size and polydispersity index indicated the adequate oil HLB.

*Nanoemulsion characterisation* - Droplet size and polydispersity index (PDI) of the nanoemulsions were determined by photon correlation spectroscopy (Zetasizer ZS, Malvern, UK). Droplet measurements were performed in triplicate and average droplet size was expressed as the mean diameter ± standard deviation.

**Fig. 1:**
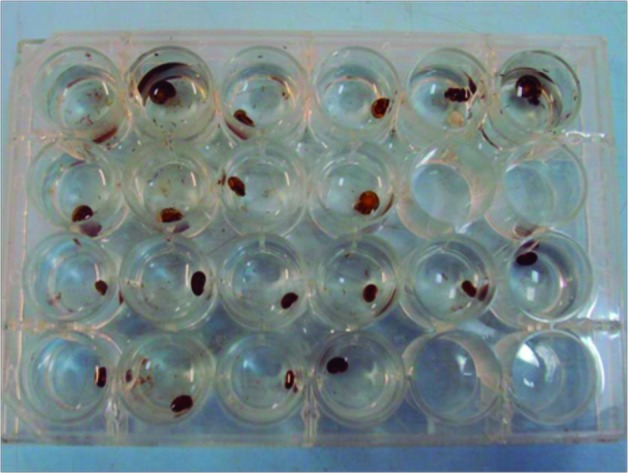
a 24-well plate divided into two groups of ten *Biomphalaria* molluscs*.*

*Molluscicidal assays* - Nanoemulsion of essential oil from *X. ochrantha* (100 ppm) was diluted to three different concentrations (80 ppm, 60 ppm, and 40 ppm) in distilled water. For the assay, 10 adult snails of *B. glabrata* species (measuring 10-12 mm diameter, from Sumidouro, RJ, Brazil) were exposed to 2 mL of the nanoemulsion, in a 24-well plate for 48 h at room temperature, according to the bioassay adapted from WHO, 1983^17^ (Fig. 1). Adult snails free of *S. mansoni* infection were kept in breeding grounds at the Laboratory for Analysis and Promotion of Environmental Health (LAPSA/IOC). Snail mortality was recorded over the following 48 h, and was compared with the nanoformulation control (formulation without oil at 100 ppm), positive control [Bayluscide WP 70® (Niclosamide) at 1 ppm], and negative control (distilled water). Absence of snail retraction into their shells after stimulation of the cephalopodal mass and/or release of haemolymph were the criteria of death. These assays were performed in triplicate.

**Fig. 2:**
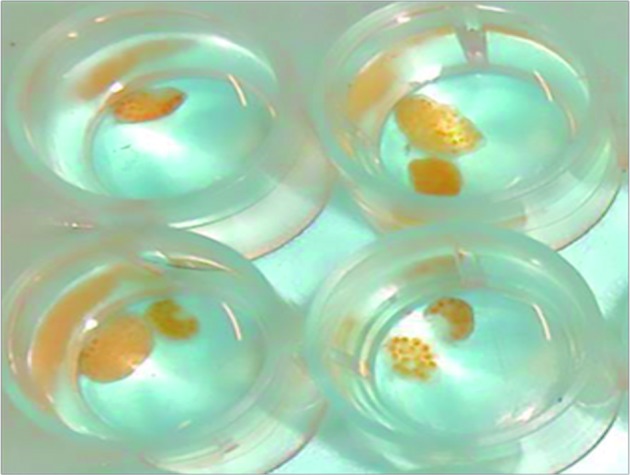
egg postures of *Biomphalaria* species in extended size.

Complementary assessments of mortality and inhibition of posture development were performed with *B. straminea*, *B. tenagophila*, and *B. glabrata* from other locations (Ressaca, Belo Horizonte, MG, Brazil). For each analysis of mortality, according to a previous protocol, 2 mL of the DL_25_, DL_50_, and DL_90_ sample concentrations obtained in the previous analysis with *B. glabrata* were used, and the groups of molluscs were divided into 10 individuals, and were separated according to size (3-5 mm, 4-6 mm, and 8-10 mm). Analysis of postures viability of *B. glabrata* (Ressaca, MG) was performed by treating them (two and five days old) with 2 mL of DL_25_, DL_50_, and DL_90_ sample concentrations, and counting the number of eggs that were not viable in relation to the initial amount, at 24 and 48 h (Fig. 2). As in the previous assay, the results were compared with the positive, negative, and nanoformulation controls.

Concentrations that killed 50% and 90% (LC_50_ and LC_90_) of the exposed snails in 24 h and 48 h (compared with the negative-control cultures) were expressed as the mean and standard deviation, and were statistically analysed using Student’s *t*-test (p ≤ 0.05), using the software R (MASS data package).

## RESULTS

*Essential oil* - Essential oil obtained from fresh leaves (2.0 g) showed a bright green colour and a yield of 0.2%. Oil density was 0.8 g/mL and pH was equal to 5.0. In total, 27 substances were identified with the predominance of sesquiterpenes and hydrocarbons (68.55%). The main substances found in these analyses were bicyclogermacrene (25.18%), and germacrene D (20.90%). Substances such as β-pinene (8.07%), sylvestrene (6.50%), and E-caryophyllene (6.23%) were also present (Table I).

**TABLE I:** Chemical composition of essential oil of leaves from *Xylopia ochrantha* (Mart.)

AI lit	AI exp	Substance	(%)
932	934	ɑ-pinene	2.7
969	974	sabinene	1.0
974	978	β-pinene	8.1
1002	1007	ɑ-phellandrene	0.7
1022	1026	o-cymene	0.9
1025	1030	sylvestrene	6.5
1032	1037	Z-β-ocimene	0.4
1044	1048	E-β-ocimene	1.3
1335	1340	δ-elemene	5.4
1374	1378	ɑ-copaene	2.3
1389	1395	β-elemene	2.4
1417	1423	E-caryophyllene	6.2
1434	1436	λ-elemene	0.5
1452	1452	cis muurola-3,5-diene	0.6
1452	1458	ɑ-humulene	0.8
1458	1465	allo-aromadendrene	0.6
1478	1481	λ-muurolene	1.0
1480	1486	germacrene D	20.9
1500	1502	bicyclogermacrene	25.2
1506	1505	ɑ-bisabolene	0.4
1522	1528	δ-cadinene	0.9
1559	1562	germacrene B	1.4
1577	1586	spathulenol	4.0
1590	1591	globulol	0.8
1592	1600	viridiflorol	0.5
1649	1659	β-eudesmol	0.7
1652	1663	ɑ-cadinol	0.5
		Total identified	96.6
			
		Monoterpeneshydrocarbons	21.6
		Sesquiterpeneshydrocarbons	68.6
		Oxygenatedsesquiterpenes	6.5

*Nanoemulsion* - The droplet size and PDI were used to select the HLB of the NE (Table II). The formulation with a HLB of 9.26 (40% of Tween 20 and 60% of Span 80), showed values of droplet size as 114 ± 1 nm and a PDI of 0.1 ± 0.0, which in turn, are characteristic of a stable nanoemulsion. The selected NE exhibited a translucent and homogeneous appearance.

**TABLE II:** Size droplet, polydispersity index (PDI) and hydrophilic-lipophilic balance (HLB) values of eleven formulations with essential oil from *Xylopia ochrantha*

Formulation	Sizedroplet (nm)	PDI	HLB
F1	329 ± 19	0.3 ± 0.2	16.70
F2	232 ± 4	0.3 ± 0.0	15.46
F3	168 ± 3	0.3 ± 0.0	14.22
F4	142 ± 3	0.2 ± 0.0	12.98
F5	112 ± 1	0.3 ± 0.0	11.74
F6	114 ± 1	0.2 ± 0.0	10.50
F7	114 ± 1	0.1 ± 0.0	9.26
F8	187 ± 1	0.3 ± 0.0	8.02
F9	227 ± 4	0.2 ± 0.0	6.78
F10	386 ± 13	0.2 ± 0.0	5.54
F11	323 ± 2	0.3 ± 0.0	4.30

Molluscicidal assays

*Effects on B. glabrata juvenile and adults from Sumidouro and Ressaca regions* - Molluscicidal activity was tested in juveniles (size of 3-5 mm), adults with 6-7 mm, and adults with 8-10 mm. Additionally, these specimens were collected from two regions, Sumidouro (RJ, Brazil) and Ressaca (MG, Brazil).

Lethal concentrations obtained from *B. glabrata* (Sumidouro, RJ) were used to test against *B. glabrata* (Ressaca, MG) of different age/size, and against other *Biomphalaria* species. Additionally, the same lethal concentrations were used in oviposition tests on *Biomphalaria*.

Nanoemulsion (NE) of the essential oil from *X. ochrantha* leaves exhibited molluscicidal activity against adult (size of 10-12 mm) *B. glabrata* (Sumidouro, RJ) with LC_50_/24 h = 50.91 ± 3.06 ppm, LC_90_/24 h = 85.46 ± 4.96 ppm, LC_50_/48 h = 47.41 ± 2.85 ppm, and LC_90_/48 h = 78.48 ± 4.35 ppm. Furthermore, in the complementary assay with *B. glabrata* (Ressaca, MG), NE was tested at three concentrations 32, 47, and 78 ppm. Among juvenile molluscs with a size of 3-5 mm, 96.7% and 100% mortality was observed at the concentrations of 47 ppm (LC_90_/48 h) and 78 ppm (LC_50_/48 h), respectively, within 24 h. The NE could cause a higher mortality rate among adults of size 6-7 mm (100% at the concentration of 47 ppm). When it was used at a concentration of 32 ppm, the NE caused mortality in 96.7% adults from this region. Adult molluscs (between 8-10 mm) were affected to a lesser extent, exhibiting mortality of 3.3% in 24 h at the concentration of 47 ppm (Table III).

**TABLE III:** Mortality index (in percentage) of different *Biomphalaria* species in different sizes/ages treated with the nanoemulsionated essential oil from *Xylopia ochrantha* in 24 h

mollusks	32 ppm	47 ppm	78 ppm	Positive control	Nano control	Negative control
	(%) mortality (24 h)					
*B. straminea* (3-5 mm)	-	90	100	100	0	0
*B. tenagophila* (3-5 mm)	-	100	100	100	0	0
*B. glabrata* (3-5 mm)	-	96.7	100	100	10	10
*B. straminea* (6-7 mm)	93.3	100	-	100	0	0
*B. tenagophila* (6-7 mm)	96.7	100	-	100	0	0
*B. glabrata* (6-7 mm)	96.7	100	-	100	0	0
*B. straminea* (8-10 mm)	-	46.7	-	100	0	0
*B. tenagophila* (8-10 mm)	-	26.7	-	100	0	0
*B. glabrata* (8-10 mm)	-	3.3	-	100	0	0

NE treatment for 48 h basically maintained the results observed after 24 h (Table IV) for all sizes of *B. glabrata* (Ressaca, MG) tested.

**TABLE IV:** Mortality index (in percentage) of different *Biomphalaria* species in different sizes/ages treated with the nanoemulsionated essential oil from *Xylopia ochrantha* in 48 h

mollusks	32 ppm	47 ppm	78 ppm	Positive control	Nano control	Negative control
	(%) mortality (48 h)					
*B. straminea* (3-5 mm)	-	93.3	100	100	0	0
*B. tenagophila* (3-5 mm)	-	100	100	100	0	0
*B. glabrata* (3-5 mm)	-	96.7	100	100	10	20
*B. straminea* (6-7 mm)	100	100	-	100	0	0
*B. tenagophila* (6-7 mm)	96.7	100	-	100	0	0
*B. glabrata* (6-7 mm)	96.7	100	-	100	0	0
*B. straminea* (8-10 mm)	-	100	-	100	0	0
*B. tenagophila* (8-10 mm)	-	53.3	-	100	0	0
*B. glabrata* (8-10 mm)	-	10	-	100	0	0

*Effects on B. straminea adults* - As in the evaluations on *B glabrata* (Ressaca, MG), NE caused mortality in molluscs of 3-5 mm in the range of 100% and 90% in 24 h when used at concentrations of 78 and 32 ppm, respectively. Adults of size 6-7 mm exhibited higher mortality of 100% at the concentration of 47 ppm and 93.3% at the concentration of 32 ppm, within 24 h. NE at the concentration of 47 ppm was evaluated in molluscs of 8-10 mm, which caused mortality in the range of 46.7% at 24 h.

**Fig. 3:**
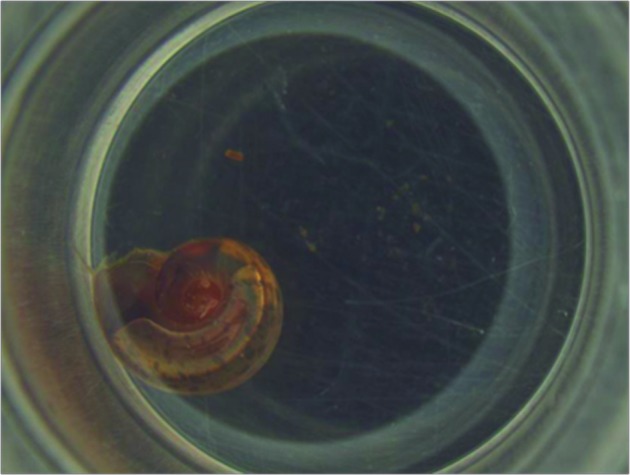
*Biomphalaria straminea* in distilled water after 48 h (Photo: LRNEM/IOC).

**Fig. 4:**
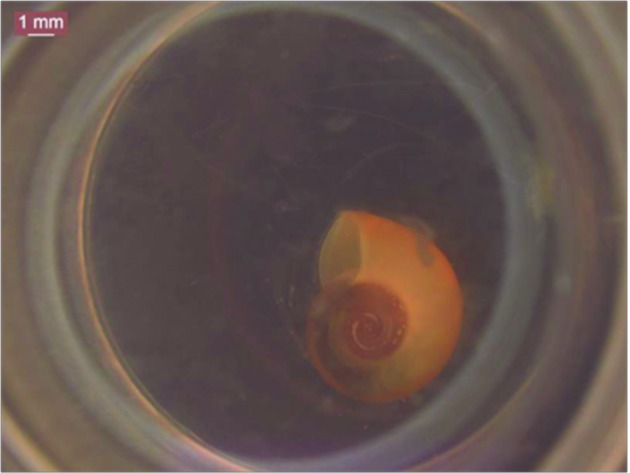
*Biomphalaria straminea* dead in contact with 47 ppm nanoemulsion (NE) after 48 h (Photo: LRNEM/IOC).

NE treatment for 48 h in molluscs of 3-5 mm treated with 78 and 32 ppm caused 100%, and 93.3% mortality, respectively. When treated for 48 h, molluscs of 6-7 mm showed 100% mortality in the presence of 47 ppm NE (Figs 3,4). Molluscs of 8-10 mm showed mortality in the range of 100% when treated with 47 ppm (Table IV).

*Effects on B. tenagophila adults* - At the highest concentration tested (78 ppm), molluscs of 3-5 mm were totally affected after 24 h, with 100% mortality. Molluscs of 6-7 mm also showed 100% mortality at 24 h, when tested at the 47 ppm concentration. In this same period, 32 ppm NE caused 96.7% mortality. In larger molluscs (8-10 mm), 47 ppm NE caused a mortality rate of 26.7% in 24 h (Table III).

**Fig. 5:**
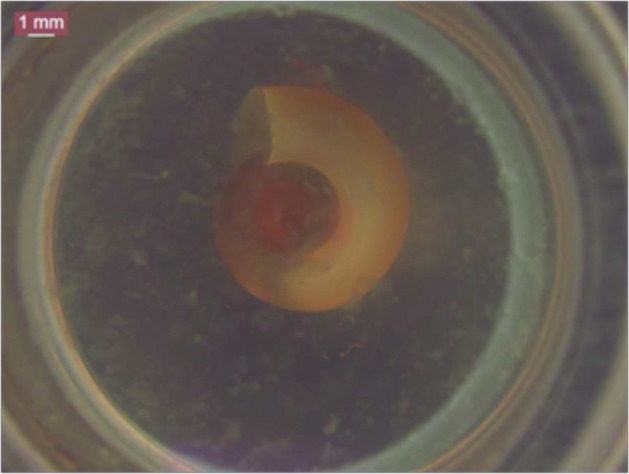
*Biomphalaria tenagophila* dead in contact with 47 ppm nanoemulsion (NE) after 48 h (Photo: LRNEM/IOC).

NE treatment for 48 h in molluscs of 3-5 mm and 6-7 mm caused 100% mortality in 47 ppm (Fig. 5). In molluscs of 8-10 mm, the concentration of 47 ppm resulted in 53.3% mortality (Tables IV).

In all these analyses, the positive control was fully effective for the three species in all sizes. NE free of essential oil did not cause mortality.

*Effects on eggs posture development* - In the analysis of eggs posture, NE was able to render the eggs from recent oviposition (two days) unfeasible in a period of 24 h with 100% inhibition for *B. straminea*, *B. tenagophila*, and *B. glabrata* (Ressaca, MG) at both concentrations (47 and 72 ppm) tested (Table V).

The five days old oviposition treated with 47 ppm NE showed 91.7% unviable eggs after 24 h of treatment in *B. straminea* molluscs. At the concentration of 78 ppm, a reduction of the effect in *B. straminea* (17.3%) was observed. The molluscs *B. tenagophila* treated with 47 ppm exhibited 49.4% unviable eggs, and 78 ppm caused a greater effect with 82.5% unviable eggs in 24 h.

**TABLE V:** Inhibition of eggs development index (in percentage) of different species of *Biomphalaria* (two and five days old) after contacting with the nanoemulsionated essential oil from *Xylopia ochrantha* in 24 h

mollusks	47 ppm	78 ppm	Positive control	Nano control	Negative control
	(%) unviable eggs (24 h)				
*B. straminea* (two days old)	100	100	100	6.9	0
*B. tenagophila* (two days old)	100	100	100	0	0
*B. glabrata* (two days old)	100	100	83.3	2	0
*B. straminea* (five days old)	91.7	17.3	100	0	0
*B. tenagophila* (five days old)	49.4	82.3	100	0	0
*B. glabrata* (five days old)	62.7	73.5	100	0	0

The molluscs *B. glabrata* (Ressaca, MG) showed 62.7% unviable eggs with 47 ppm, and 73.5% with 78 ppm for the same egg stage and time (Table V).

In 48 h, all species eggs at two days were rendered unfeasible (Table VI). When the eggs were observed at late oviposition (five days), NE caused the maximal effect on *B. straminea* and *B. glabrata* (Ressaca, MG). In late oviposition, *B. tenaghofila* at 47 ppm and 78 ppm showed 70.4% and 95.2% unviable eggs, respectively.

**TABLE VI:** Inhibition of eggs development index (in percentage) of different species of *Biomphalaria* (two and five days old) after contacting with the nanoemulsionated essential oil from *Xylopia ochrantha* in 48 h

mollusks	47 ppm	78 ppm	Positive control	Nano control	Negative control
	(%) unviable eggs (48 h)				
*B. straminea* (two days old)	100	100	100	6.9	0
*B. tenagophila* (two days old)	100	100	100	45	0
*B. glabrata* (two days old)	100	100	100	2	0
*B. straminea* (five days old)	100	100	100	0	0
*B. tenagophila* (five days old)	70.4	95.2	100	0	0
*B. glabrata* (five days old)	100	100	100	0	0

## DISCUSSION

Bicyclogermacrene and germacrene D, two major substances of the leaf essential oil from *X. ochrantha*, are the main components in other species of the genus such as *X. langsdorffiana* A.St.-Hil. & Tul., *X. aethiopica* (Dunal) A.Rich., and *X. aromatica* (Lam.) Mart. Furthermore, high occurrence of sesquiterpenes in foliar essential oil is also observed in these species.^6^ ^,^ ^18^ ^,^ ^19^ The yield of leaf essential oil (0.20%) is similar to that in other species of the genus, in a range from 0.07% and 0.43%, relative to fresh leaves.^19^ ^,^ ^20^ ^,^ ^21^ Bicyclogermacrene is best known for its antimicrobial and fungicidal activities.^22^ ^,^ ^23^ ^,^ ^24^ Germacrene D is cited in literature as a simulator of insect pheromones, and is also involved in other important mechanisms of the insect-plant relationship.^25^ ^,^ ^26^ Additionally, a previous study demonstrated molluscicidal activity from the leaf essential oil of *X. langsdorffiana* against *B. glabrata*, in which, germacrene D was the major compound.^6^ This result can suggest an association with the activity found in the essential oil from *X. ochrantha* against *B. glabrata*, since these species are included in the same genus and have germacrene D as one of the two main constituents.

Although the constituents of essential oils from *X. ochrantha* are related to relevant biological activities, they have low solubility in water, which hinders their dispersion into the environment in aquatic media. Thus, delivery in aqueous carriers as nanoemulsions (NEs) is required. NEs were successfully produced using a low energy method of spontaneous emulsification. This method is simple and only uses magnetic stirring.^8^ The aqueous phase was dripped over the organic phase under continuous magnetic stirring for 1 h. The transition of surfactants from the oil phase to aqueous phase produces interfacial turbulence and spontaneous formation of nanometric oil droplets that are involved and stabilised by surfactants^27^ (Table II). The following criteria were used for the analysis and selection of the formulation: size < 200 nm; PDI < 0.25; and organoleptic characteristics: absence of phase separation and precipitation. The formulation with HLB of 9.26 (40% of Tween 20 and 60% of Span 80) showed low values of droplet size and polydispersity index, which in turn, are characteristic of a nanoemulsion system. Development of a system to provide a suitable nanoformulation with a size below 200 nm for essential oil from *X. ochrantha* allows a more efficient aqueous dispersibility, stability, and delivery of actives in the target of action. NEs as delivery systems for essential oils permit interaction between the active principles and biological membranes. In general, this occurs through one of four main routes: 1-increased surface area and passive transport across the plasma membrane of cells; 2-fusion of the droplets with the cell membrane occurring specifically upon delivery of the substances at the site of action; 3-reservoir effect with sustained release of essential oil; 4-electrostatic interaction between positively charged droplets and negatively charged biological membrane favouring bioadhesion and biological effects.^8^ These facts highlight the relevance of bionanotechnology as an effective alternative for the control of diseases through inhibition of intermediate hosts of parasites present in the environment. Moreover, the biodegradability of the formulation is another factor that minimises residual toxicity problems of molluscicidal agents to the environment.

Molluscicidal activity of the nanoemulsion containing the essential oil from *X. ochrantha* against *Biomphalaria* molluscs could be observed in all evaluated species, including individuals of different ages/sizes and at different stages of egg development. According to the observed effects on mortality of *B. glabrata* of size 10-12 mm (Sumidouro, RJ), the LC_90_/24h (85.46 ppm) of the oil is considered satisfactory, as the value recommended by the WHO for a plant sample to be considered active is DL_90_/48h < 100 ppm.^3^ Differences in action on individuals of different species could be observed, so that comparing the NE concentration (47 ppm) at 24h, *B. tenaghofila* appeared to be more susceptible to NE, though all species with sizes of 3-5 mm and 6-7 mm presented a high mortality rate. Adult individuals of *B. glabrata* (8-10 mm) exhibited resistance. Treatment for 48 h at the concentration of 47 ppm caused an effect similar to that at 24 h. In contrast to old specimens (8-10 mm) that increased the susceptibility at *B. straminea*.

Another observation can be noted when comparing the sizes of individuals within the same species, wherein smaller/younger molluscs were found to be less resistant to NE action, suggesting that older individuals may have a more developed resistance system against the active components of NE.

NE also acts on oviposition with an inhibitory effect at the concentrations of 47 ppm and 78 ppm. All eggs, from more recent oviposition (two days), presented 100% mortality in 24 h. In eggs from older oviposition (five days), 100% inhibition of development was observed only in a period of 48 h, although inhibition was also observed at 24 h, at varying degrees. These results suggest a higher resistance to oil compounds by the more developed eggs of *B. tenaghofila* species.

Finally, these biological assays demonstrated the molluscicidal activity of the nanoemulsified essential oil from *X. ochrantha* and the inhibition of different stages of development in *Biomphalaria* molluscs, which denotes the biological importance of a species with poor scientific information regarding controlling disease transmitters.

*In conclusion* - This study reported the molluscicidal activity and chemical composition of essential oil from leaves of *X. ochrantha*, a Brazilian endemic species. This is the first report on the activity of this species in the control of molluscs that are part of infectious diseases cycles, more specifically against *Biomphalaria* that are involved in the transmission of schistosomiasis. Thus, this result suggests the use of nanoemulsified essential oil from *X. ochrantha* as a promising alternative for *Biomphalaria* control, as this plant species is endemic to a country affected by schistosomiasis.
